# Standing sagittal alignment of the whole axial skeleton with reference to the gravity line in humans

**DOI:** 10.1111/joa.12586

**Published:** 2017-01-27

**Authors:** Kazuhiro Hasegawa, Masashi Okamoto, Shun Hatsushikano, Haruka Shimoda, Masatoshi Ono, Takao Homma, Kei Watanabe

**Affiliations:** ^1^ Niigata Spine Surgery CenterNiigataJapan; ^2^ Department Of Orthopaedic SurgeryNiigata University Medical and Dental HospitalNiigataJapan

**Keywords:** aging, force plate measurement, gravity line, sagittal whole body alignment, standing balance

## Abstract

Human beings stand upright with the chain of balance beginning at the feet, progressing to the lower limbs (ankles, knees, hip joints, pelvis), each of the spinal segments, and then ending at the cranium to achieve horizontal gaze and balance using minimum muscle activity. The details of the alignment and balance of the chain, however, are not clearly understood, due to the lack of information regarding the three‐dimensional (3D) orientation of all bony elements in relation to the gravity line (GL). We performed a clinical study to clarify the standing sagittal alignment of whole axial skeletons in reference to the GL using the EOS slot‐scanning 3D X‐ray imaging system with simultaneous force plate measurement in a healthy human population. The GL was defined as a vertical line drawn through the centre of vertical pressure measured by the force plate. The present study yielded a complete set of physiological alignment measurements of the standing axial skeleton from the database of 136 healthy subjects (a mean age of 39.7 years, 20–69 years; men: 40, women: 96). The mean offset of centre of the acoustic meati from the GL was 0.0 cm. The offset of the cervical and thoracic vertebrae was posterior to the GL with the apex of thoracic kyphosis at T7, 5.0 cm posterior to the GL. The sagittal alignment changed to lordosis at the level of L2. The apex of the lumbar lordosis was L4, 0.6 cm anterior to the GL, and the centre of the base of the sacrum (CBS) was just posterior to the GL. The hip axis (HA) was 1.4 cm anterior to the GL. The knee joint was 2.4 cm posterior and the ankle joint was 4.8 cm posterior to the GL. L4‐, L5‐ and the CBS‐offset in subjects in the age decades of 40s, 50s and 60s were significantly posterior to those of subjects in their 20s. The L5‐ and CBS‐offset in subjects in their 50s and 60s were also significantly posterior to those in subjects in their 30s. HA was never posterior to the GL. In the global alignment, there was a positive correlation between offset of C7 vertebra from the sagittal vertical axis (a vertical line drawn through the posterior superior corner of the sacrum in the sagittal plane) and age, but no correlation was detected between the centre of the acoustic meati‐GL offset and age. Cervical lordosis (CL), pelvic tilt (PT), pelvic incidence, hip extension, knee flexion and ankle dorsiflexion increased significantly with age. Our results revealed that aging induces trunk stooping, but the global alignment is compensated for by an increase in the CL, PT and knee flexion, with the main function of CL and PT to maintain a horizontal gaze in a healthy population.

## Introduction

The human skeleton in standing position is considered like a ‘reverse pendulum’, with the chain of balance beginning at the support polygon (both feet), progressing to the lower limb skeleton (ankles, knees, hip joints, pelvic vertebra), the spinal segments, and finally to the cephalic vertebra working as a pendulum to achieve horizontal vision and balance. All of these elements work in concert to maintain erect posture, a characteristic of humans, for which the ‘cone of economy’ represents perfect balance requiring a minimum of muscle activity in normal situations (Dubousset, 1994). Human beings are able to stand still and walk upright due to the lordotic lumbar curvature and concomitant alignment of the upper spine, lordotic cervical spine, kyphotic thoracic spine and the appropriate pelvis position connecting to the lower extremities. Once alignment is decompensated forward, however, malalignment leads to a decrease in the health‐related quality of life (Glassman et al. 2005). Itoi (1991) investigated the relationship between the sagittal posture of the spine and the lower extremities in osteoporotic subjects, and found that thoracic kyphosis, a primary deformity, appears to be compensated for by the lumbar spine, sacroiliac joint, hip joint and knee joint. These reports suggest that not only spinal alignment, but also alignment of the lower extremities are crucial factors in standing balance. Therefore, to understand the entire mechanism of standing balance, it is important to clarify the features of precise alignment from the head to the feet. Sagittal spinal alignment in humans was recently extensively investigated using X‐ray measurements, and several important findings were reported (Stagnara et al. 1982; During et al. 1985; Bernhardt & Bridwell, 1989; Duval‐Beaupère et al. 1992; Jackson & McManus, 1994; Gelb et al. 1995; Korovessis et al. 1998; Legaye et al. 1998; Vaz et al. 2002; Duval‐Beaupère & Legaye, 2004; Berthonnaud et al. 2005; Vialle et al. 2005; Boulay et al. 2006; Le Huec & Hasegawa, 2016). Standardized data of whole skeletal alignment in the standing position, however, have not been fully investigated, probably due to the limitations of conventional X‐ray measurements in which a fan‐beam X‐ray significantly magnifies the subject in the margin of the cassette. The EOS system (EOS Imaging, Paris, France), a slot‐scanning three‐dimensional (3D) X‐ray imager, was developed by the combined efforts of multidisciplinary partners, physical radiation engineers, biomechanical engineers, medical radiologists and orthopaedic paediatric surgeons to overcome the limitations of conventional X‐ray measurement. The 3D bone external envelope technique incorporates simultaneous anteroposterior and lateral X‐rays of the whole body, making 3D reconstruction possible at every level of the osteo‐articular system and especially the spine in the standing position. The EOS allows for more precise bone reconstruction in orthopaedics, especially at the level of spine, pelvis and lower limbs, with limited X‐ray exposure (Dubousset et al. 2005; Deschenes et al. 2010). To clarify the characteristics of erect posture, the ‘cone of economy’, i.e. the 3D orientation of all the bony elements in relation to the gravity line (GL), is an important concept from a biomechanical point of view.

We hypothesized that humans stand with appropriate sagittal profile to achieve horizontal vision, and the deterioration of the spinal alignment due to aging is compensated by a supportive function of spine, pelvis and lower extremities to maintain the horizontal gaze. The purpose of this study was to test the hypothesis by investigating standing sagittal alignment of whole axial skeletons in reference to the GL using the EOS system with simultaneous force plate measurement in a healthy human population.

## Materials and methods

After institutional review board approval, 158 volunteers with no history of treatment for spinal disease were enrolled. Informed consent for the present study was obtained from all subjects. Following EOS imaging (described below), we excluded eight cases with lumbarization, five cases with sacralization, four cases with 11 thoracic vertebrae and five cases with scoliosis with a Cobb angle > 20 ° to perform accurate radiographic measurements. Exclusion of the transitional vertebrae is important because these vertebrae affect the measurement of spinal and pelvic parameters. Consequently, 136 cases with a mean age of 39.7 years (20–69 years; men: 40, women: 96) were analysed. The epidemiological and morphological characteristics of this cohort were obtained from the following data: age, sex, weight and height. The body mass index (BMI) was calculated as the weight in kilograms divided by the square of the height in metres. To assess the health‐related quality of life, we used the Japanese version of the Oswestry Disability Index (ODI; Fairbank & Pynsent, 2000; Fujiwara et al. 2003) and Scoliosis Research Society‐22 score (SRS‐22; Asher et al. 2003; Hashimoto et al. 2007). The ODI and SRS‐22 have become the principal condition‐specific outcome measures used for the management of low back disorders and spinal deformities, respectively. Normal values with no symptoms are 0 (%) in ODI and five in the SRS‐22, and the worst values are 100 (%) in the ODI and 0 in the SRS‐22.

### Radiological measurement

Routine radiographs were obtained using the EOS system (Dubousset et al. 2005; Carreau et al. 2014), and tracking of the centre of gravity using a force plate was simultaneously recorded as follows.

**Figure 1:**
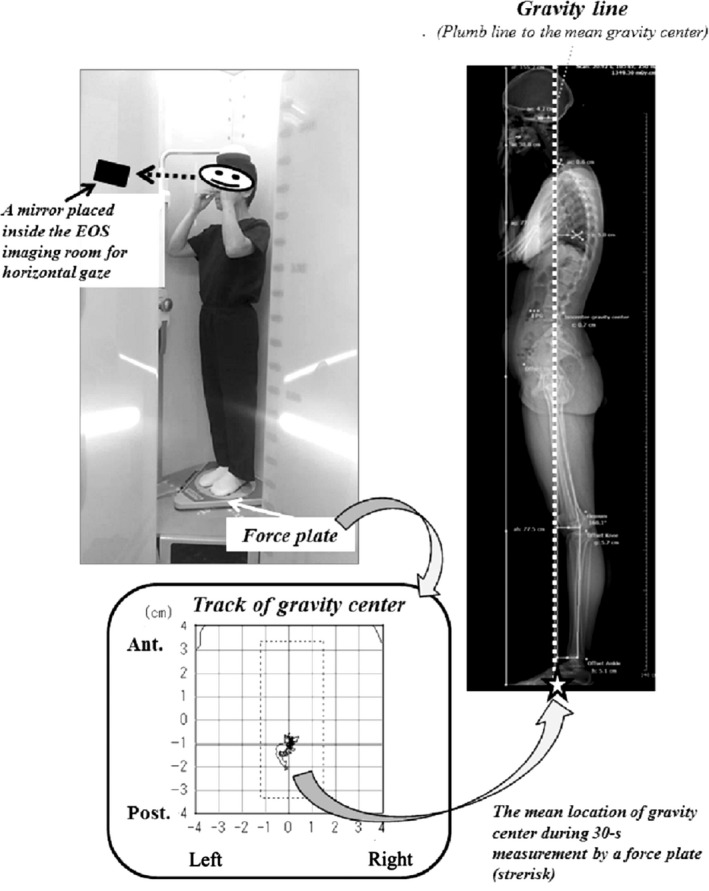
EOS imaging and the definition of the gravity line (GL) using a simultaneous force plate measurement.

1. EOS radiographs were obtained from the head, including the centre of the acoustic meati (CAM) to the feet.
2. Each patient was asked to stand barefoot comfortably on the force plate with their hands on their cheeks.
3. A mirror placed at eye level in the inner wall of the EOS box helped the patient to maintain a horizontal gaze (Fig. 1).

The default scan speed of the EOS system is 7.6 cm s^−1^. Acquisition time is linked to scan height: time of acquisition (s) = height of acquisition (cm)/7.6. Therefore, subtle artefacts in the images can occur due to body sway during scanning, but the artefacts are minimized by the rapid X‐ray detection time (0.8333 ms) with no blurring of the images.

The EOS system allows the acquisition of frontal and lateral views simultaneously, with a scanning technology that performs undistorted 1/1 scale acquisition, in a weight‐bearing position. Using the two acquisitions, anatomical landmarks are first manually identified on the pelvis, allowing the identification of the true patient sagittal plane (a truly sagittal pelvic plane) and the radiological plane. Indeed, a slight rotation of the pelvis can have an impact on the two‐dimensional (2D) measurements of the pelvis performed on lateral standard X‐ray systems. Following computation with matching the pelvis in the radiological plane to that in the patient (true pelvic) plane by rotating with 3D volume data, EOS allows measurement of all parameters in the true sagittal pelvic plane.

We measured the following parameters.

#### GL defined by force plate measurement

Tracking of the gravity centre was recorded during EOS imaging ~20 s in the transversal plane with a force plate (ANIMA, Tokyo, Japan). The mean location of the track was defined as the mean gravity centre, and the vertical line from the gravity centre as the GL (Fig. 1).

#### Cranio‐cervical alignment

**Figure 2:**
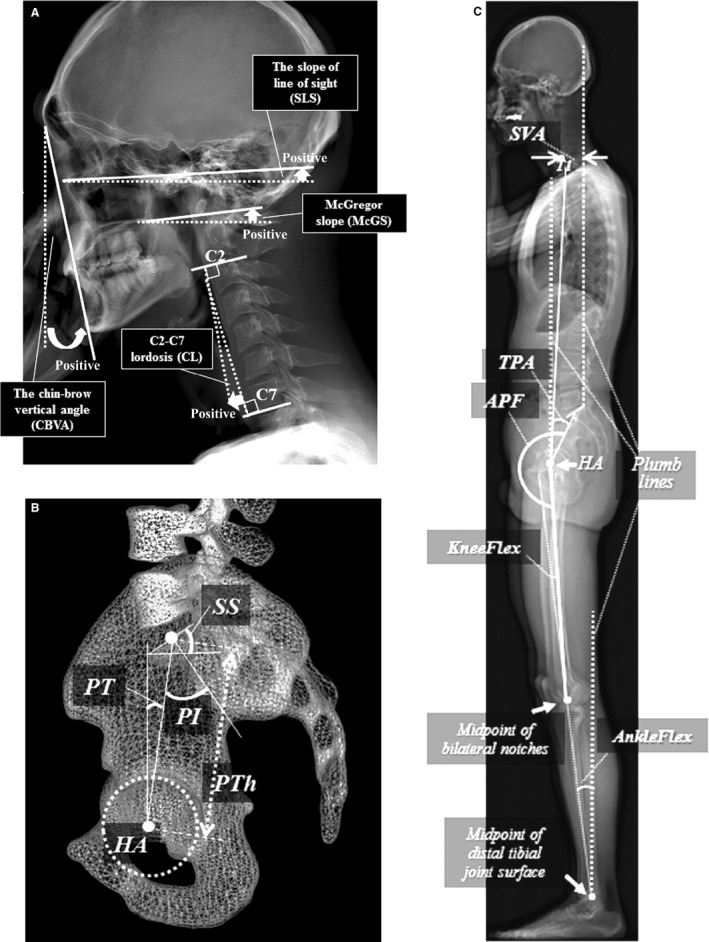
(A) Cranio‐cervical alignment. CBVA, the chin–brow vertical angle; SLS, the slope of the line of sight; McGS, McGregor slope; CL, C2–C7 lordosis. Each arrow represents a positive value (Lafage et al. 2016). (B) Sagittal pelvic parameters superimposed in the 3D reconstructed mesh modeled by EOS system. HA (hip axis), the midpoint of the line between both femoral heads; SS, sacral slope; PT, pelvic tilt; PI, pelvic incidence (= PT + SS); PTh, pelvic thickness (Le Huec et al. 2011). (C) Spinopelvic and lower extremity parameters. SVA, offset of C7 vertebra from the sagittal vertical axis (a vertical line drawn through the posterior superior corner of sacrum in the sagittal plane); TPA (T1 pelvic angle), sum of the PT and angle between the plumb line from the HA and the line from the HA to the centre of T1 (Protopsaltis et al. 2014); angle pelvi‐femoral (APF), angle between the line from the centre of the base of the sacrum (CBS) to the HA and the line from the centre of the knee joints to the HA (Mangione & Senegas, 1997); KneeFlex, mean of bilateral knee flexion angles; and AnkleFlex, mean of bilateral ankle flexion angles.

The chin–brow vertical angle (CBVA), the slope of the line of sight (SLS), McGregor slope (McGS; Lafage et al. 2016) and C2–C7 lordosis (CL) were measured (Fig. 2A).

#### Standard sagittal spino‐pelvic alignment

T1–T12 (Kyph), L1–S1 lumbar lordosis (LL), sacral slope (SS), pelvic tilt (PT), pelvic incidence (PI; Duval‐Beaupère et al. 1992; Legaye et al. 1998) and pelvic thickness (PTh; Le Huec et al. 2011) were measured as standard sagittal alignment parameters (Fig. 2B).

#### Spinopelvic and lower extremity parameters

As a consideration of global spinal alignment, the distance between the vertical plumb line from the posterior edge of the base of the sacrum and the centre of the vertebral body of C7 [sagittal vertical axis (SVA)], which has been used as a marker of true postural balance by spine surgeons (Roussouly et al. 2006; Schwab et al. 2013), the sum of the PT and the angle between the plumb line from the hip axis (HA), the midpoint of the line between both femoral heads, and the line from the HA to the centre of T1 (T1 pelvic angle; Protopsaltis et al. 2014) were measured. Hip joint extension was determined using the angle pelvi‐femoral (APF), between the line from the centre of the base of the sacrum (CBS) to the HA and the line from the centre of the knee joints to the HA (Mangione & Senegas, 1997). The mean of the bilateral knee flexion angles (KneeFlex) was defined as the angle between the line from the HA to the midpoint of the bilateral notches of the femoral condyles and the line from the notch to the midpoint of the distal tibial joint surfaces. The angle between the line from the notch to the midpoint of the distal tibial joint surfaces and the plumb line from the midpoint was defined as the ankle dorsiflexion angle (AnkleFlex; Fig. 2C).

#### Offset distance between the bony landmark and GL

**Figure 3:**
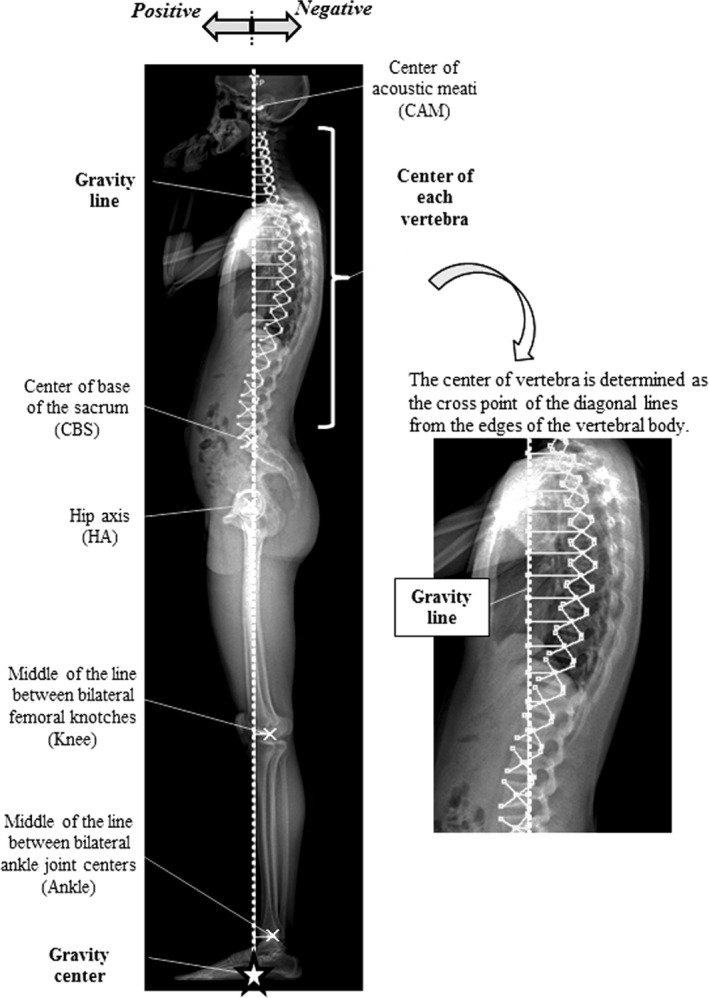
Measurement of the distance between all bony landmarks and the gravity line (GL).

The bony landmarks of the whole axial skeleton in the standing sagittal plane were determined in the lateral standing EOS image as follows (Fig. 3).

1. The centre of the head was defined as the CAM. Because the CAM can be clearly identified in a lateral X‐ray image and is located close to the gravity centre of the head, which was determined by the suspension method (Vital & Senegas, 1986).
2. The centre of all vertebral bodies, from C2 to L5, was determined as the cross‐point of the diagonal lines from the edges of the vertebral body.
3. The CBS was defined as the middle of the anterior and posterior edges of the base of the sacrum.
4. The centre of the hip joints was defined as the HA.
5. The centre of the knee joint was defined as the middle of the line between the bilateral femoral notches (Knee).
6. The centre of the ankle joint was defined as the middle of the line between the bilateral ankle joint centres (Ankle).

The distance between all the bony landmarks and GL was measured in all subjects.

### Statistical analysis

The jmp software package (version 9; SAS Institute, Cary, NC, USA) was used for all statistical analyses. A *P‐*value of less than 0.05 was considered statistically significant.

1. Mean, 95% confidence limits and the values of the interquartile range (25% and 75%) were calculated for all demographic and radiographic parameters.
2. Subjects were divided into five age groups by decade: 20s (*n* = 27); 30s (*n* = 46); 40s (*n* = 35); 50s (*n* = 20) and 60s (*n* = 8). Differences among age groups and health‐related quality of life (ODI/SRS‐22) and the offset of all the bony landmarks were analysed. The values of ODI score were not normally distributed, thus non‐parametric comparisons with Steel–Dwass analysis for all the pairs were performed. The comparisons in SRS‐22 (normally distributed in all the age groups) were analysed with one‐way anova and Tukey–Kramer's HSD analysis.
3. Simple linear regression analyses were performed to estimate the effect of age among the radiographic parameters.

## Results

### Demographic and radiographic parameters

Mean values with standard deviation (SD), standard error (SE) and the lower/higher 95% confidence interval of all the demographic and radiological standard parameters are reported in Table 1. The distributions of all the parameters, except ODI, were normal. There were significant differences in PT, PTh, SVA, APF and KneeFlex between men and women (Table 2).

**Table 1:** Demographic and basic sagittal spinal alignment of the subjects (*n* = 136, 40 male/96 female)

	Mean	95% Confidence intervals	Interquartile range, 25%/75% values
Age (years)	39.7	37.8/41.6	31.3/47.0
Men (years)	40.0	36.3/43.7	32.0/48.3
Women (years)	39.6	37.4/41.8	31.0/47.0
BMI^a^	21.4	20.9/21.9	19.5/23.1
ODI^b^ (%)	5.1	4.0/6.2	0/8.2
SRS‐22^c^	4.3	4.3/4.4	4.1/4.5
CBVA^d^ (°)	5.4	4.4/6.4	2.0/9.5
SLS^e^ (°)	0.5	−0.2/1.7	−3.1/5.5
McGS^f^ (°)	3.8	2.8/4.8	−0.4/8.1
CL^g^ (°)	−3.1	−5.0/−1.3	−10.9/4.5
T1–12 kyphosis (°)	41.8	40.1/43.4	34.5/48.7
L1–S1 LL (°)	55.5	53.6/57.4	49.3/62.7
SS (°)	40.7	39.3/42.2	36.0/46.1
PT (°)	11.3	10.0/12.6	6.4/15.7
PI (°)	52.0	50.2/53.9	44.8/60.1
PTh (cm)	10.9	10.8/11.0	10.4/11.4
SVA^h^ (cm)	0.0	−0.4/0.4	−1.6/1.6
TPA^i^ (°)	15.4	14.0/16.8	10.4/20.3
APF^j^ (°)	196.7	195.4/198.0	190.7/202.3
KneeFlex^k^ (°)	−1.6	−2.3/−0.8	−4.8/1.8
AnkleFlex^l^ (°)	4.0	3.6/4.4	2.3/5.7

a Body mass index (BMI) was calculated as the weight in kilograms divided by the square of the height in metres (kg m^−2^).

b The Oswestry Disability Index (ODI; Fairbank & Pynsent, 2000).

c Scoliosis Research Society‐22 (SRS‐22; Asher et al. 2003).

d The chin–brow vertical angle (CBVA; Lafage et al. 2016)

e Slope of the line of sight (SLS; Lafage et al. 2016).

f McGregor slope (McGS; Lafage et al. 2016).

g C2–C7 lordosis (CL).

h The offset between the vertical line through the posterior edge of the base of the sacrum and the centre of the vertebral body of C7 (positive means stooping; Roussouly et al. 2006; Schwab et al. 2013).

i T1 pelvic angle. Sum of T1 inclination on the HA and PT (Protopsaltis et al. 2014).

j Angle pelvi‐femoral (APF). The angle formed by the middle of the S‐1 endplate and HA, and the line between HA and the femoral axis. The range in an asymptomatic population was 191 ± 7 ° (Mangione & Senegas, 1997).

k Average flexion angle of the bilateral knees. Negative number indicates extension and positive number indicates flexion.

l Average angle between the line from the mid‐point of the bilateral femoral notches and that of the distal tibial joints, and the plumb line. Negative number indicates plantar flexion and positive number indicates dorsi‐flexion.

LL, lumbar lordosis; PI, pelvic incidence; PT, pelvic tilt; PTh, pelvic thickness; SS, sacral slope.

**Table 2:** Comparison of the values of alignment parameters (mean ± SD) between men (*n* = 40) and women (*n* = 96)

Parameters	Men	Women	*P*‐value
CBVA^a^(°)	5.3 ± 5.2	5.4 ± 6.0	0.8127
SLS^b^ (°)	0.5 ± 4.6	0.8 ± 6.2	0.7170
McGS^c^ (°)	4.1 ± 4.6	3.7 ± 6.5	0.9848
CL^d^ (°)	−0.6 ± 8.6	−4.2 ± 11.6	0.0546
Kyph^e^ (°)	43.7 ± 9.0	41.0 ± 10.2	0.2689
LL^f^ (°)	56.4 ± 12.7	55.1 ± 10.3	0.6813
SS^g^ (°)	40.9 ± 9.6	40.7 ± 7.8	0.8597
PT^h^ (°)	9.2 ± 6.2	12.2 ± 7.8	0.0257
PI^i^ (°)	50.1 ± 11.2	52.9 ± 10.7	0.0652
PTh^j^ (cm)	10.7 ± 0.8	11.0 ± 0.7	0.0360
SVA^k^ (cm)	−0.6 ± 2.3	0.3 ± 2.3	0.0368
TPA^l^ (°)	13.5 ± 7.6	16.2 ± 8.6	0.0855
APF^m^ (°)	193.3 ± 6.6	198.1 ± 8.1	0.0005
KneeFlex^n^ (°)	−0.3 ± 4.5	−2.1 ± 4.4	0.0438
AnkleFlex^o^ (°)	4.1 ± 2.3	4.0 ± 2.3	0.8429

Comparison by Wilcoxon's rank sum test

a The chin–brow vertical angle (CBVA).

b The slope of the line of sight (SLS).

c McGregor slope (McGS; Lafage et al. 2016).

d C2–C7 lordosis (CL; positive means lordosis).

e T1–T12 kyphosis (kyph).

f L1–S1 lumbar lordosis (LL).

g Sacral slope (SS).

h Pelvic tilt (PT).

i Pelvic incidence (PI; Duval‐Beaupère et al. 1992; Legaye et al. 1998).

j Pelvic thickness (PTh; Le Huec et al. 2011).

k The offset between the vertical line through the posterior edge of the base of the sacrum and the centre of the vertebral body of C7 (positive means stooping; Roussouly et al. 2006; Schwab et al. 2013).

l The sum of the PT and the angle between the plumb line from the hip axis (HA; Protopsaltis et al. 2014).

m Hip joint extension determined using the angle pelvi‐femoral (APF; Mangione & Senegas, 1997).

n The mean of the bilateral knee flexion angles (positive means flexion).

o The ankle dorsiflexion angle measured between the line from the notch to the midpoint of the distal tibial joint surfaces and the plumb line from the midpoint.

The mean ODI and SRS‐22 was 5.1% and 4.3%, respectively. The mean ODI in all age groups was less than 10%. Fairbank & Pynsent (2000) reported that the mean ODI score in the normal population is 10.2%. Thus, the cohort in the present study can be regarded as normal. The mean value of SRS‐22 score (4.3) was comparable to the normal value (4.26) from healthy adolescents from 10 to 19 years old (*n* = 3052) reported recently (Daubs et al. 2014). The mean ODI and SRS‐22 values in each age group were 20s: 4.2 and 4.4; 30s: 4.3 and 4.3; 40s: 6.1 and 4.4; 50s: 5.5 and 4.2; and 60s: 8.0 and 4.3. There was no statistically significant difference among the age groups in either measure (Table 3).

**Table 3:** ODI score and SRS‐22 according to age group

Sub‐scales	20s (*n* = 27)	30s (*n* = 46)	40s (*n* = 35)	50s (*n* = 20)	60s (*n* = 8)
ODI^a^ (%)(mean ± SD)	4.2 ± 5.9	4.3 ± 4.7	6.1 ± 8.4	5.5 ± 5.5	8.0 ± 8.9
95% CI	1.9/6.5	2.9/5.7	3.1/9.1	2.8/8.1	0/16.2
SRS‐22^b^ (mean ± SD)	4.4 ± 0.3	4.3 ± 0.4	4.4 ± 0.3	4.2 ± 0.4	4.3 ± 0.3
95% CI	4.2/4.4	4.2/4.4	4.2/4.5	4.0/4.4	4.0/4.6

Steel–Dwass analyses for all the pairs in ODI (not normally distributed) were performed. The comparisons in SRS‐22 (normally distributed) were analysed with one‐way anova and Tukey–Kramer's HSD analysis. There was no significant difference among the age groups.

a The Oswestry Disability Index (ODI) score.

b Scoliosis Research Society‐22 (SRS‐22); subtotal score.

### Offset distance between the bony landmarks and GL

**Figure 4:**
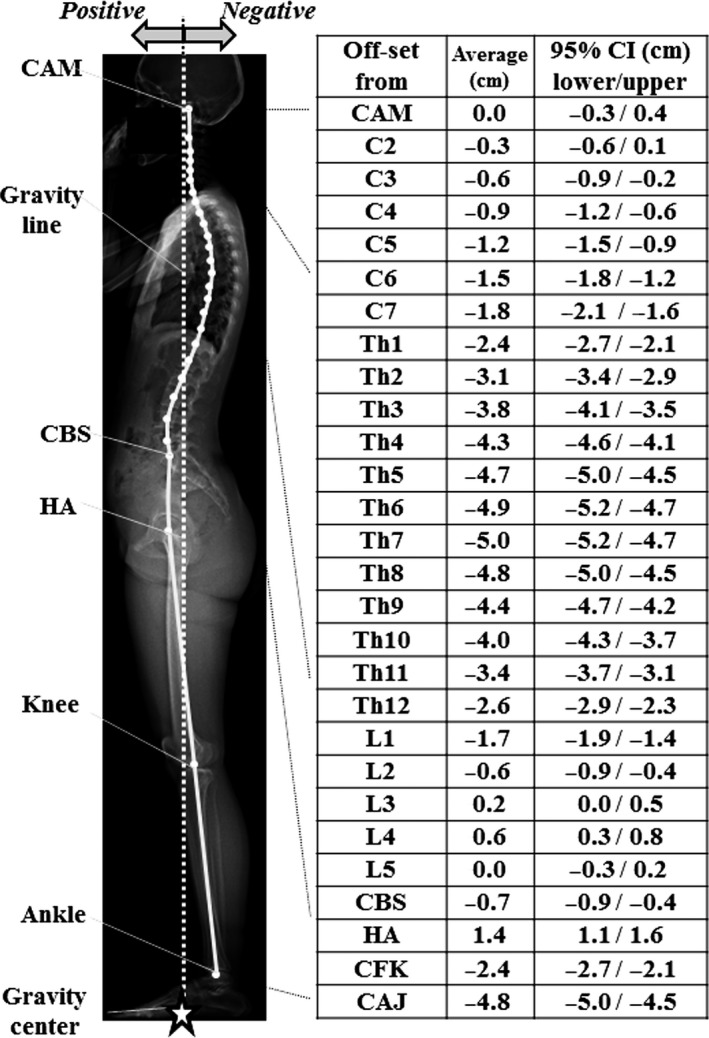
Average and 95% confidence interval of offset between all the landmarks and the gravity line (GL). All the bony landmarks are indicated as dots in the figure. Dots with no explanation denote the centre of each vertebral body.

The mean value of the CAM offset was 0. The mean offset of the cervical and thoracic vertebrae was posterior to the GL with the apex of thoracic kyphosis at T7, 5 cm posterior to the GL. The sagittal alignment changed to lordosis at the level of L2. The apex of the LL was L4, 0.6 cm anterior to the GL, and the CBS was just posterior to the GL. HA was 1.4 cm anterior to the GL. The knee was 2.4 cm posterior and the ankle was 4.8 cm posterior to the GL (Fig. 4).

### Age‐related difference in the offset of the bony landmarks

**Figure 5:**
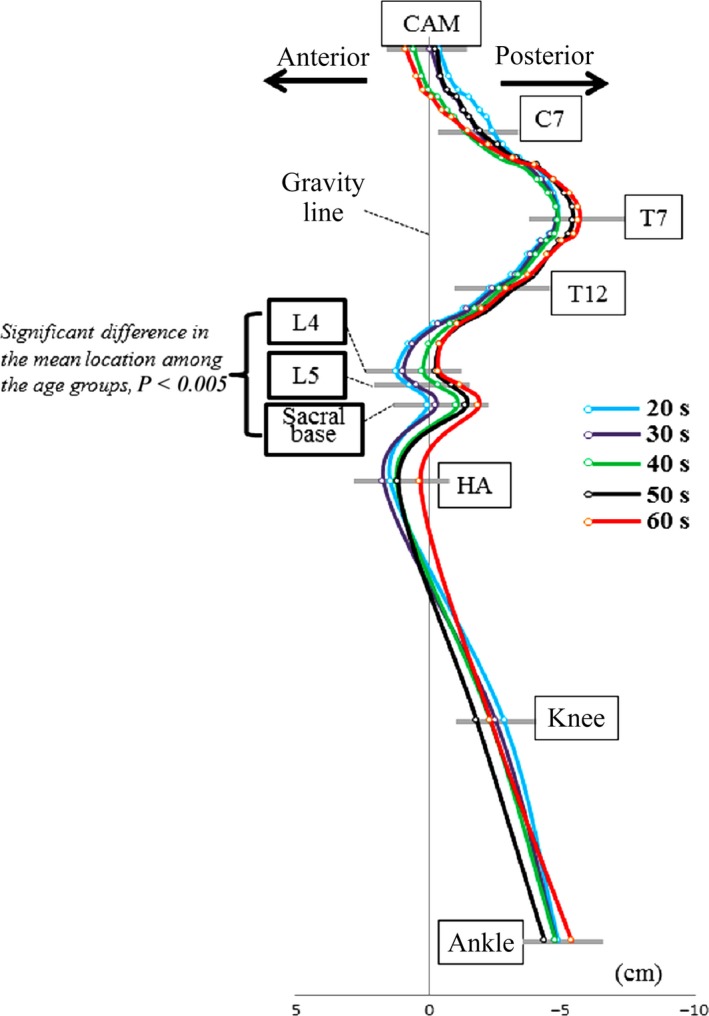
Mean location of bony landmarks according to age group with reference to the gravity line (GL).

The L4‐, L5‐ and CBS‐offset in the groups of subjects in their 40s, 50s and 60s were significantly posterior to those of subjects in their 20s. The L5‐ and CBS‐offset in subjects in their 50s and 60s were also significantly posterior to those of subjects in their 30s. The tendency was the same in the location of T7 and HA among age groups, but the mean location of the HA was never behind the GL. At all the other levels, there was no significant difference between the offset and age groups (Fig. 5).

### Simple linear regression analyses of age and the radiographic parameters

In the global alignment, SVA and TPA were positively correlated with age (*P* < 0.0001, *P* < 0.01, respectively), but no correlation was detected between CAM‐GL‐offset and age. With increasing age, the following angles and parameters all increased significantly: CL (*P* < 0.01); kyph (*P* < 0.05); PT (*P* < 0.01); PI (*P* < 0.05); APF (*P* < 0.05); KneeFlex (*P* < 0.01); AnkleFlex (*P* < 0.05; Table 4).

**Table 4:** Result of simple linear regression analyses among the measured parameters and age

Parameters	Decision coefficient (*r* ^2^)	*P*‐value	Slope of the regression line	Intercept of the regression
CBVA^a^	0.0058	0.3910	−0.0400	6.9720
SLS^b^	0.0069	0.3388	−0.0441	2.4807
McGS^c^	0.0095	0.2598	−0.0532	5.9153
CL^d^	0.1058	0.0001	0.3225	−15.9544
Kyph^e^	0.0457	0.0125	0.1918	34.1563
LL^f^	0.0093	0.2631	−0.0968	59.3261
SS^g^	0.0004	0.8218	−0.0148	41.3323
PT^h^	0.0945	0.0003	0.2085	3.0202
PI^i^	0.0385	0.0220	0.1936	44.3585
PTh^j^	0.0068	0.3381	−0.0054	11.1312
SVA^k^	0.1368	<0.0001	0.0781	−3.0971
TPA^l^	0.0562	0.0055	0.1797	8.2725
APF^m^	0.0394	0.0206	0.1430	191.0203
KneeFlex^n^	0.0621	0.0034	0.1008	−5.5798
AnkleFlex^o^	0.0341	0.0313	0.0384	2.4860

a The chin–brow vertical angle (CBVA).

b The slope of the line of sight (SLS).

c McGregor slope (McGS; Lafage et al. 2016).

d C2–C7 lordosis (CL).

e T1–T12 kyphosis (kyph).

f L1–S1 lumbar lordosis (LL).

g Sacral slope (SS).

h Pelvic tilt (PT).

i Pelvic incidence (PI; Duval‐Beaupère et al. 1992; Legaye et al. 1998).

j Pelvic thickness (PTh; Le Huec et al. 2011).

k The offset between the vertical line through the posterior edge of the base of the sacrum and the centre of the vertebral body of C7 (Roussouly et al. 2006; Schwab et al. 2013).

l The sum of the PT and the angle between the plumb line from the hip axis (HA; Protopsaltis et al. 2014).

m Hip joint extension determined using the angle pelvi‐femoral (APF; Mangione & Senegas, 1997).

n The mean of the bilateral knee flexion angles.

o The ankle dorsiflexion angle measured between the line from the notch to the midpoint of the distal tibial joint surfaces and the plumb line from the midpoint.

## Discussion

Humans stand and walk with regulated neuromuscular control of the base of the skeleton. The axial skeleton, the chain of balance, should be aligned under the balance of the ‘cone of economy’ (Dubousset, 1994). Standing spinal curvature fundamentally correlates with the pelvic anatomy, especially with PI (Duval‐Beaupère et al. 1992). Based on the stability and mobility of the spino‐pelvic structure, the trunk balances above the lower extremities. Although standing sagittal alignment has been extensively investigated, the alignment of whole axial skeleton in reference to the GL has never been investigated. In the present study, we clarified the relationship between the GL and representative bony landmarks in standing whole axial skeletons using the EOS imaging system. Furthermore, we strictly excluded all subjects with anomalous vertebrae, including suspected transitional vertebrae and scoliosis with a Cobb angle > 20 °, which can affect the precision of measurement. Thus, the data in this study are considered accurate. The present study yields a physiological human standard for several spino‐pelvic parameters (Table 1) and these are similar to those reported by previous authors (Stagnara et al. 1982; During et al. 1985; Bernhardt & Bridwell, 1989; Duval‐Beaupère et al. 1992; Jackson & McManus, 1994; Gelb et al. 1995; Korovessis et al. 1998; Legaye et al. 1998; Vaz et al. 2002; Duval‐Beaupère & Legaye, 2004; Vialle et al. 2005; Boulay et al. 2006; Le Huec & Hasegawa, 2016). A large PI is associated with a greater SS and a pronounced LL, and a low PI is associated with a smaller SS and a subtle LL, leading to a basic concept of ‘équilibre économique’ during standing (Dubousset, 1994; During et al. 1985; Duval‐Beaupère et al. 1992; Legaye et al. 1998; Duval‐Beaupère & Legaye, 2004; Roussouly & Pinheiro‐Franco, 2011).

When spinal balance, i.e. dynamic stability, is considered, it is necessary to define the GL to determine the muscle activity. Several studies have been performed using force plate measurements (Gangnet et al. 2003; El Fegoun et al. 2005; Schwab et al. 2006; Lafage et al. 2008; Mac‐Thiong et al. 2009; Steffen et al. 2010). An increased risk of a poor ODI (> 34) was observed in patients with a C7 plumb line greater than 6 cm, a GL greater than 6 cm and a C7 plumb line anterior to the GL (Mac‐Thiong et al. 2009). Normal spinal balance in the standing position has yet to be clarified. We were able to determine the location of full body bony landmarks in the standing position in reference to the GL for the first time (Fig. 4). The results are comparable with previous fragmental results (Duval‐Beaupère & Legaye, 2004; Gagnet et al. 2003; Steffen et al. 2010). Especially, the description of the relationship in the present study is almost identical to that of Duval‐Beaupère's group, which was deduced from the study of barycentremetry and computer simulation (Duval‐Beaupère & Legaye, 2004). Therefore, we consider that the complete set of whole body alignment measurements from a healthy population in the present study could be referred to as the normative value of humans including gender difference in PT, PTh, SVA, APF and KneeFlex (Table 2).

**Figure 6:**
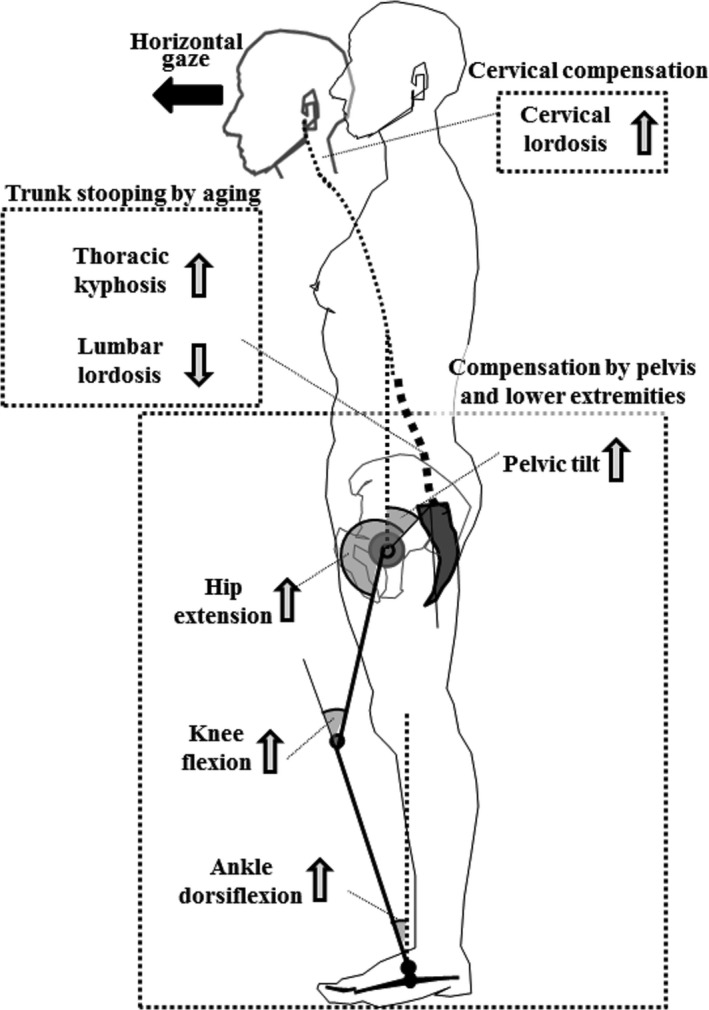
Compensation mechanism to maintain horizontal gaze. Aging induces trunk stooping, but the global alignment is compensated for by an increase in the cervical lordosis (CL), pelvic tilt (PT) and knee flexion (KneeFlex), with CL and PT being the main mechanisms of compensation in a healthy population.

Cervical lordosis, Kyph, PT, PI, APF, KneeFlex and AnkleFlex increased with age. Regarding global alignment, SVA was positively correlated with age, but CAM‐GL was not positively correlated with age (Table 4). CAM‐GL was close to 0 through the age decades of the 20s to the 60s, suggesting that the final purpose of standing posture, a horizontal gaze (Vital & Senegas, 1986), was realized in healthy subjects. The L4, L5 vertebrae and sacrum shifted posteriorly with age (Fig. 5). A posterior shift of the lower lumbar vertebrae and sacrum is considered to induce the loss of lordosis and an increase in the PT. Roussouly hypothesized three stages of compensation in progressive kyphosis: (i) the normal situation with a slight pelvis retroversion and C7 plumb line over the sacral endplate; (ii) a compensated stage, with a progressive loss of lordosis and pelvic retroversion to maintain the C7 PL behind the femoral heads; and (iii) a decompensated stage, hip extension limits the pelvic retroversion that is compensated for by flexion of the knees, but the C7 plumb line passes forward to the femoral heads (Roussouly & Pinheiro‐Franco, 2011). Adult spinal deformity patients with spinopelvic mismatch present with pelvic retroversion and flattening of thoracic kyphosis, and become exhausted with increasing mismatch, at which point there seems to be a steady transfer of compensation toward significant participation of the lower limbs (Diebo et al. 2015). Our results are compatible with the hypothesis in the past articles that aging induces trunk stooping, but the global alignment is compensated for by an increase in the CL, PT and knee flexion. The decision coefficient of the CL (*r* ^2^ = 0.1058) and PT (*r* ^2^ = 0.0945) was, however, greater than that of the Kneeflex (*r* ^2^ = 0.0621; Table 4), suggesting that CL and PT play a greater role in the healthy population (Fig. 6).

Pelvic incidence is a crucial parameter that determines the whole standing sagittal alignment. Mangione et al. (1997) measured PI on radiographs of 30 foetuses, 30 children and 30 adults, and found that PI considerably increases during the first few months of life, continues to increase during the early years of life, and stabilizes at about 10 years old. On the other hand, Tardieu et al. (2013) investigated pelvic morphology in 50 adults and 19 intact neonates, and found that sacro‐acetabular distance and PI are negatively correlated, and conversely PI and LL are positively correlated, suggesting that the two linkages developed simultaneously during hominid evolution (Tardieu et al. 2013). Although PI has been believed to be a consistent value after completion of growth, PI increased about 10 ° on average from 20 to 70 years old in the present study (Table 4). Vrtovec et al. (2012) reviewed PI values from 47 papers, and concluded that PI tends to increase with age in normal and scoliotic subjects. We assume that the alteration of PI with age is caused by an increase of movement of the sacroiliac joint, the only site possibly affecting PI value, due to osteoarthritic change. The assumption is, however, in contradiction with the previous literatures (Mangione et al. 1997; Marty et al. 2002; Diebo et al. 2015). Therefore, the authors cannot conclude that PI increases with age, and we have to continue to seek a relationship between PI and age with close attention to the sacroiliac joint.

In conclusion, measurement of standing sagittal alignment of whole axial skeletons in reference to the GL using the EOS system with simultaneous force plate measurement in a healthy human population yielded a complete set of data regarding the physiological alignment of the standing axial skeleton. The mean offset of CAM from the GL was 0. The offset of cervical and thoracic vertebrae was posterior to the GL with the apex of thoracic kyphosis at T7, 5 cm posterior to the GL. Sagittal alignment changed to lordosis at L2. The apex of the LL was L4, 0.6 cm anterior to the GL, and CBS was just posterior to the GL. HA was 1.4 cm anterior to the GL. The knee joint was 2.4 cm posterior and the ankle joint was 4.8 cm posterior to the GL. The L4‐, L5‐ and CBS‐offsets in subjects in their 40s, 50s and 60s were significantly posterior to those of subjects in their 20s. The L5‐ and CBS‐offsets in subjects in their 50s and 60s were also significantly posterior to those of subjects in their 30s. HA was never behind the GL. In the global alignment, there was a positive correlation between SVA and age, but no correlation between CAM‐GL‐offset and age. CL, PT, PI, hip extension, knee flexion and ankle dorsiflexion were positively correlated with age. These results support the hypothesis of the present study that humans stand with appropriate sagittal profile to achieve horizontal vision, and the deterioration of the spinal alignment due to aging is compensated by a supportive function of spine, pelvis and lower extremities to maintain the horizontal gaze.

## Disclosures

None of the authors has received any grant or financial support for the present study.
